# Subcontinuum mass transport of condensed hydrocarbons in nanoporous media

**DOI:** 10.1038/ncomms7949

**Published:** 2015-04-22

**Authors:** Kerstin Falk, Benoit Coasne, Roland Pellenq, Franz-Josef Ulm, Lydéric Bocquet

**Affiliations:** 1 Department of Civil and Environmental Engineering and MultiScale Material Science for Energy and Environment UMI 3466 CNRS-MIT, Massachusetts Institute of Technology, 77 Massachusetts Avenue, Cambridge, Massachusetts 02139, USA

## Abstract

Although hydrocarbon production from unconventional reservoirs, the so-called shale gas, has exploded recently, reliable predictions of resource availability and extraction are missing because conventional tools fail to account for their ultra-low permeability and complexity. Here, we use molecular simulation and statistical mechanics to show that continuum description—Darcy's law—fails to predict transport in shales nanoporous matrix (kerogen). The non-Darcy behaviour arises from strong adsorption in kerogen and the breakdown of hydrodynamics at the nanoscale, which contradict the assumption of viscous flow. Despite this complexity, all permeances collapse on a master curve with an unexpected dependence on alkane length. We rationalize this non-hydrodynamic behaviour using a molecular description capturing the scaling of permeance with alkane length and density. These results, which stress the need for a change of paradigm from classical descriptions to nanofluidic transport, have implications for shale gas but more generally for transport in nanoporous media.

## Abstract (web summary)

Shale gas and oil are trapped in nanoscale porous networks of ultra-low permeability. Here, the authors develop a molecular model of alkane transport through nanoporous materials, showing that the mechanisms controlling flow at the nanoscale lead to a simple scaling of permeance with hydrocarbon size and density.

## Graphical abstract

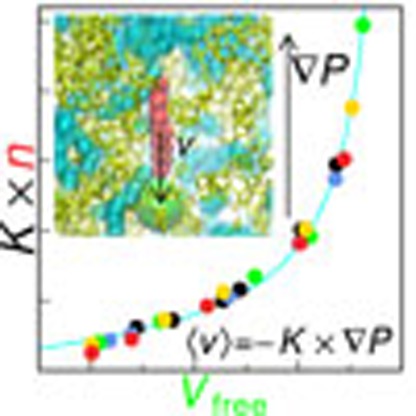

Over the last decade, natural gas recovery from shales has increased worldwide, particularly in the United States, where production rates are skyrocketing—nowadays about 40% of the natural gas produced in the United States, as compared with 1% in 2000 (refs 1, 2). Predictions foresee this transformation to continue with part of the attention shifted to shale oil. However, the reliability of these predictions is highly disputed^3^,^4^ because of large uncertainties over the availability of this resource and large concerns about its environmental impact^5^,^6^.

From a scientific perspective, shale gas and oil are trapped in a complex network of small pores, in particular in organic inclusions (kerogen) with sub-nanometre pore space^7^. A key characteristic of these unconventional reservoirs is their ultra-low permeability^8^. Quantitatively, flow rate predictions are classically based on Darcy's law


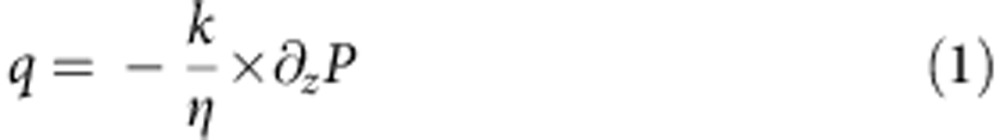


stating that the volumetric fluid flux through a porous material depends linearly on the pressure gradient, the inverse of the fluid viscosity *η* and a material-specific permeability *k*. Typically, the permeability scales as the square of the pore diameter and is measured in Darcy (1D≃0.987 × 10^−12^ m^2^). Unconventional reservoirs exhibit permeabilities of the order of 10^−9^ D, typically six orders of magnitude smaller than conventional reservoirs^8^, and in direct line with the nanoporous structures of kerogen^7^. Considering that kerogen is the hydrocarbon source, which produces the gas and oil through its decomposition, the slow and complex hydrocarbon migration from kerogen to the cracks surface is the rate-limiting step^9^,^10^,^11^. Such ultra-low permeability raises concerns on the applicability of the Darcy framework itself to account for mass transport in the nanoporous kerogen. Although attempts have been made to palliate for the breakdown of Darcy approach by including slippage in gas flow, via, for example, the Klinkenberg effect^12^,^13^, such empirical corrections cannot capture the complex adsorption and transport behaviour of hydrocarbon in ultra-confining porous materials. Such effects must manifest themselves through a complex interplay between apparent viscosity and wettability, as evidenced in recent experiments on nanoconfined water^14^.

At a more global scale, some recent works aimed at explaining the specific longtime production rates of shales beyond traditional reservoir modelling. Monteiro *et al*.^9^ suggested a hydrodynamic model of gas flow in nanoporous media by introducing a pressure gradient-dependent permeability of kerogen. They predict a power law for the decline of the production rate, which is compatible with early-life data for several major US shale plays. In the same line, Patzek *et al*.^10^ proposed a simplified model of shale reservoirs, which are made up of parallel equidistant fracture planes. Assuming Darcy-like gas flow bewteen these fracture planes, they predict a crossover from an early-time algebraic decay to an exponential decline at long time. Although such macroscale modellings capture some specificities of the gas recovery, in particular the long-time decay, they, however, point to the lack of knowledge on small-scale behaviours, and in particular on the role of the adsorption and desorption processes, as well as non-Darcy multiphase flow. Further research is needed to improve the—so far limited—scientific understanding^2^.

As far as the fundamental question of fluid transport in nanoporous materials is concerned, one expects two major reasons for the breakdown of the Darcy framework. First, strong adsorption effects occurring in nanopores are expected to induce large changes in the phase behaviour of the confined hydrocarbons^15^,^16^. The density of the alkane phase inside the nanoporous material is usually much larger than its bulk counterpart and confined hydrocarbons are expected to behave as a condensed phase, at odd with the simple gas picture. This has potentially dramatic consequences for their transport properties^17^,^18^,^19^. Second, research in the field of ‘nanofluidics' has demonstrated the breakdown of hydrodynamics at the nanoscale^20^,^21^,^22^; new phenomena such as slippage, interfacial transport and non-viscous effects appear as the ‘molecular granularity' of the fluid becomes non-negligible. Overall, hydrocarbon transport in the multiscale and disordered nanoporosity of kerogen remains essentially not understood. Keeping in mind that large parts of the total amount of hydrocarbons is trapped in this nanoporosity, and that the overall permeability of the formation will be limited by the lowest permeability in the fluid path, there is a strong need for a reliable theoretical framework of hydrocarbon transport in nanoporous matrix, with the ultimate goal of obtaining more reliable predictions, towards a more efficient and environmentally safe exploitation technology.

Here, we present an in-depth theoretical study of *n*-alkane transport in a kerogen-like nanoporous matrix, which aims at proposing such a new theoretical framework. By relying on statistical mechanics molecular simulations that capture the interplay between adsorption and transport as well as the breakdown of hydrodynamics at the nanoscale, our approach does not require assuming any flow type (Darcy, diffusive, Knudsen and so on). We first show that the continuum description—the so-called Darcy's law—dramatically fails to describe transport within a molecular model of nanoporous kerogen. Such a failure of the conventional description is shown to be due to the non-viscous nature of the flow in such complex media, which arises from strong alkane adsorption. Nevertheless, despite the intrinsic complexity of such heterogeneous, disordered media, all permeances are shown to follow an unexpected yet simple scaling with the alkane length. To account for the scaling of permeance with alkane length and fluid density, we propose a molecular description in which transport arises from a combination of slip-like friction of the hydrocarbons with the matrix and a free volume term. This model provides an analytical expression for the permeance, which allows to rationalize hydrocarbon transport in kerogen and quantitatively describe the permeance for all alkanes, at all densities.

## Results

### Alkane transport in kerogen

**Figure 1:**
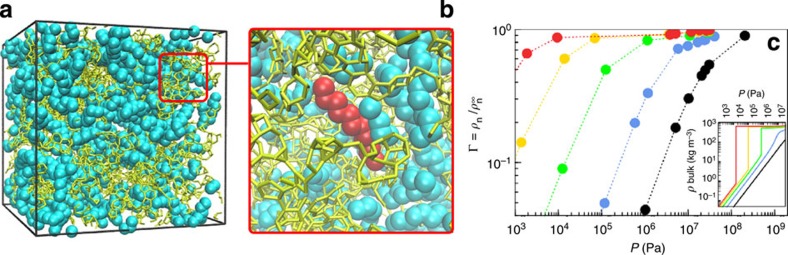
Hydrocarbons in kerogen-like nanoporous carbon under reservoir conditions. (**a**) System setup: *n*-alkanes adsorbed in a porous carbon matrix (volume (5 nm)^3^); (**b**) zoom on one dodecane molecule (red) with its neighbours and the surrounding carbon structure; (**c**) adsorption isotherms of methane (black), propane (blue), hexane (green), nonane (yellow) and dodecane (red), normalized by the maximum density 
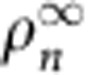
 reached at high pressures; the mass density 
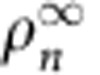
 increases slightly with the alkane length (see Supplementary Methods and Supplementary Fig. 2). Because of the small pore sizes (∼1 nm), the system is dominated by fluid/solid interfaces, and the fluid is in a supercritical phase, that is, no gas–liquid phase transition occurs. *Inset:* bulk phase diagrams for comparison.

Figure 1a,b shows the nanoporous structure used for this study; a disordered porous carbon, obtained using an atom-scale reconstruction technique, which can be seen as a reasonable molecular model of kerogen as it captures its main features (pore size, density, chemical composition including sp^2^/sp^3^ hybridization ratio, morphological disorder)^23^,^24^,^25^,^26^. The pore size distribution of the numerical sample considered here spans from a few Å to ∼15 Å, which is fully consistent with the pore sizes probed by N_2_ and CO_2_ adsorption in kerogen (see Supplementary Fig. 1 for a comparison with available experimental pore size distributions). We investigated both hydrocarbon adsorption and transport in this molecular model of kerogen using configurational biased grand-canonical Monte Carlo and molecular dynamics simulations. Details about the models and simulations can be found in the Supplementary Discussion and Methods; see also Supplementary Table 1. This will serve as the basis of a theoretical scaling model of transport, based on the analysis of the fluctuations of microscopic variables via the fluctuation dissipation theorem (FDT)^27^. Such a bottom-up approach will allow us to assess fluid transport in ultra-low permeable materials on the relevant microscopic scale (Fig. 1).

**Figure 2:**
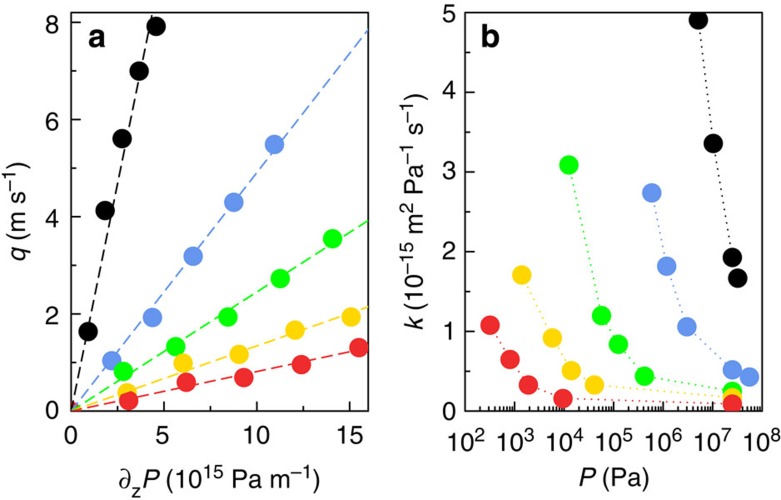
Alkane transport in kerogen-like nanoporous carbons. Flow of different *n*-alkanes in nanoporous carbon under an external driving force −∇*P*: methane (black), propane (blue), hexane (green), nonane (yellow) and dodecane (red). (**a**) Linear response of the mean flow velocity to the pressure gradient (*T*=423 K, *P*=25 MPa, dashed lines: linear fits); (**b**) permeance *K*=−*q*/∇*P* as a function of the thermodynamic equilibrium pressure *P*. Values of the permeance *K* are in full agreement with *equilibrium* calculation based on Green–Kubo relationship, see Supplementary Fig. 5a. This demonstrates that the linear relationship obtained here pertains to small pressure drops relevant to experimental conditions.

Adsorption and transport of linear alkanes—methane, propane, hexane, nonane and dodecane—in the kerogen-like nanoporous carbon^28^ shown in Fig. 1 were investigated under temperature and pressure relevant to shale reservoir conditions (*T*=423 K and *P*≤100 MPa). Here, we present the key results from an extensive investigation of adsorption, diffusion and steady-state flow under constant pressure gradients. As shown in Fig. 2a, the mean fluid flow velocity *q* in the matrix depends linearly on the pressure gradient ∂_*z*_*P* for all considered *n*-alkanes and static pressures,


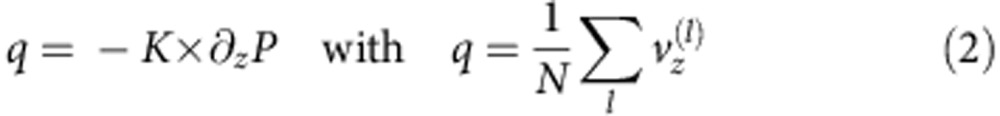


(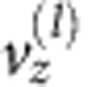 is the velocity of molecule *l*, *l*∈{1;*N*}). We emphasize that this linear relation is in no way imposed, but is a result of the simulations. In other words, no nonlinear effects occur. Note that we checked that the values of the permeance *K* are in full agreement with *equilibrium* calculation based on Green-Kubo relationship, see Supplementary Fig. 5a. This demonstrates that the linear relationship obtained here pertains to the small pressure drops relevant to experimental conditions. However, the proportionality factor *K*—called *permeance* to make a clear distinction from the *permeability k*∼*K* × *η* usually defined by Darcy's law (equation (1))—depends on the fluid type and the thermodynamic conditions as shown in Fig. 2b.

### Non-Darcy behaviour and transport scaling law

**Figure 3:**
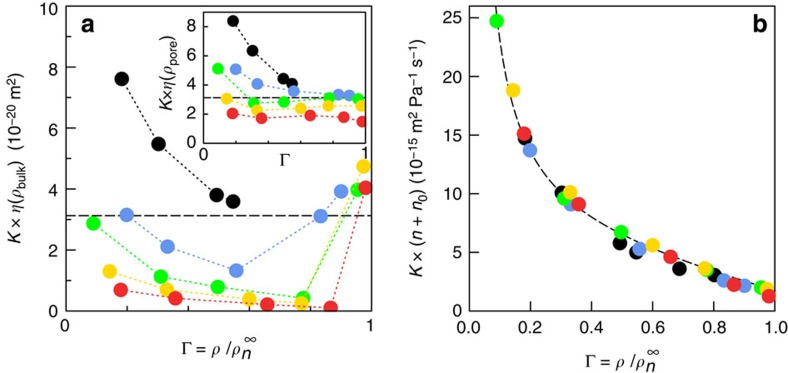
Breakdown of Darcy law and permeance master curve for alkane transport. (**a**) Permeability *η* × *K* versus loading Γ 
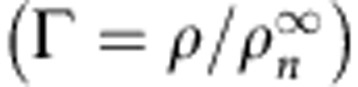
, showing the breakdown of the hydrodynamic prediction for the permeance: methane (black), propane (blue), hexane (green), nonane (yellow) and dodecane (red). The viscosity is that of the bulk hydrocarbon at the corresponding pressure and temperature. Inset: Same plot with the bulk viscosity replaced by the bulk viscosity calculated at the relevant pore density, *ρ*_pores_. For comparison, the dashed lines give the permeability of a cylindrical pore with diameter equal to the mean size of the matrix pore-size distribution. (**b**) Permeance master curve: *K* × (*n*+*n*_0_) (with *n*_0_=2) versus loading (same symbols as in **a**). This demonstrates that *K*∼1/*n* with *n* the alkane length. The dashed line is a guide to the eye.

When using Darcy's law, it is implicitly assumed that the permeability *k* is an *intrinsic material* property, that is, *k*∼*K* × *η* is a constant depending only on the geometry of the porous matrix. Figure 3a shows that this expectation dramatically fails for hydrocarbon transport in kerogen as *k* is found to depend on both the fluid type and adsorbed amount. A first reason for this failure of the classical porous-media-flow description can be found in the adsorption behaviour. As seen from the form of the adsorption isotherms in Fig. 1c, owing to the severe confinement in small nanopores such as in kerogen, the confined alkanes are in a state that drastically differs from their bulk counterpart at the same pressure and temperature^15^,^16^. In particular, comparison with the bulk phase shows that longer alkanes are in a condensed liquid-like phase under confinement while they are in a gaseous phase in bulk. As a result, the use of the bulk viscosity in this case is clearly inappropriate to calculate flow properties in the nanopores. In an attempt to extend Darcy's law to hydrocarbon transport in nanoporous media, we compared its predictions against the data in Fig. 3 when using the bulk viscosity of the alkanes at the density of the confined phase (Supplementary Fig. 3). As shown in the inset in Fig. 3a, Darcy's law with such corrected viscosities also fails to describe the permeabilities observed in the molecular simulations. We emphasize that such a pure dynamical effect cannot be accounted for by the so-called ‘Darken factor', which describes the thermodynamic effect of adsorption on transport by correcting local density gradients using local adsorption isotherms^29^.

To further assess the magnitude of the hydrodynamic breakdown, many insights are provided by the molecular dynamics. An interesting probe of the dynamical processes is the transverse momentum fluctuations, defined in Fourier space:


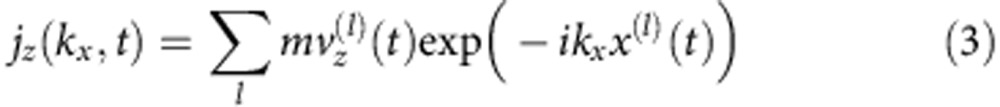


(with *x* and *z* two perpendicular directions). In a viscous fluid, transverse momentum relaxes via momentum diffusion and its correlation should exhibit a ‘universal' exponential decay at small *k* and long times^27^: 〈*j*_*z*_(*k*,*t*)*j*_*z*_(−*k*,0)〉_*equ*_∼exp(−*k*^2^*vt*) (*ν*=*η*/*ρ* is the kinematic viscosity). When confined inside a solid matrix, a viscous relaxation of the form exp[−(*γ*_0_+*k*^2^*ν*)*t*] may be expected, with *γ*_0_ steming from the Darcy friction of the liquid with the solid matrix. In strong contrast, we find a very different behaviour for the transverse momentum fluctuation of the confined alkanes, with a correlation in the form of a double exponential 〈*j*_*z*_(*k*,*t*)*j*_*z*_(−*k*,0)〉_*equ*_=*A* exp(−*a*_*k*_*t*)−*B* exp(−*b*_*k*_*t*), and a complex dependence of the decay coefficients *a*_*k*_ and *b*_*k*_ on the wave vector *k* (Supplementary Fig. 4). These features demonstrate the violation of the hydrodynamic relaxation for all explored *k* scales. It suggests that non-local effects and memory effects as described in generalized hydrodynamics with Mori-Zwanzig memory functions may occur^30^. This result shows unambiguously that alkane transport in disordered nanoporous materials such as kerogen cannot be accounted for, *at any length scale explored*, by a hydrodynamic description.

### Scaling law and nanofluidic transport

The failure of the hydrodynamic approach under extreme confinement therefore calls for alternative frameworks of alkane transport in kerogen. A lead is suggested in Fig. 3b where it is shown that, in spite of this complexity, permeances *K* for all alkanes can be collapsed onto a single master curve as a function of loading 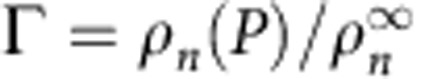 (ratio of the alkane density to its value at very large pressure, where the adsorbed amount reaches a plateau. The maximum density 
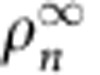
, which was obtained from a Langmuir fit of the adsorption isotherms shown in Supplementary Fig. 2, slightly depends on the alkane length *n*. The permeance *K* is found to scale as the inverse of the alkane length (number of carbon atoms *n*):


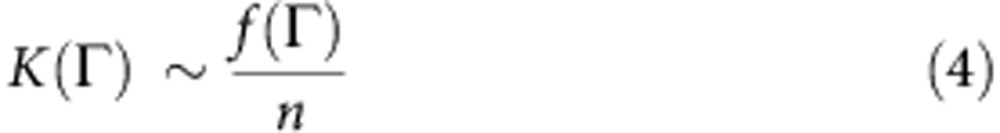


where *f*(Γ) is a simple function of the loading Γ. More specifically, we find that 
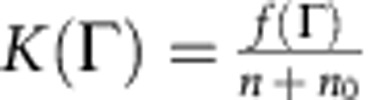
 with *n*_0_≈2 for all alkanes provides an excellent rescaling.

With the aim to propose a molecular model of alkane transport in disordered nanoporous materials such as kerogen, we make use of the intimate links between dissipation and fluctuation of microscopic quantities, as described by the FDT^27^. In this framework, the permeance *K* is expressed in terms of the fluctuation of the total momentum via a Green–Kubo equation:


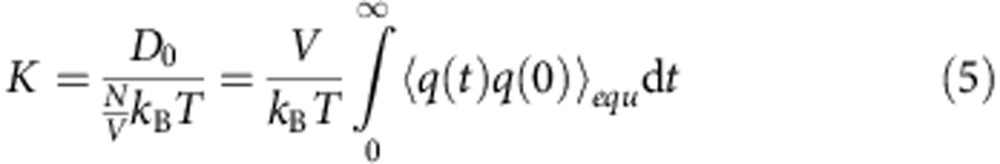


where *D*_0_ is the collective diffusion coefficient, *N* the number of alkane molecules, *V* the volume of the matrix, respectively, and 
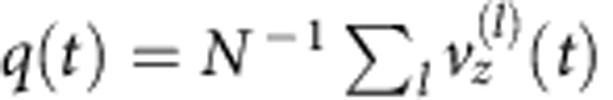
 the fluctuating centre-of-mass velocity of the fluid with respect to the frozen matrix. As expected from the FDT described in equation (5), the collective diffusivity *D*_0_ for the different confined alkanes—computed using equilibrium molecular dynamics (MD) of the *q*-autocorrelation function—is in full agreement with the permeances *K* estimated using non-equilibrium MD, in which the flow is induced by a pressure gradient (Supplementary Fig. 5a). This results further confirms that hydrocarbon transport in kerogen is in the linear regime over the entire range of pressure gradients considered. Owing to its collective nature, *D*_0_ differs from the molecular self-diffusivity *D*_*s*_ by cross-correlation terms (
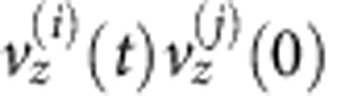
 with *i*≠*j*) of the form


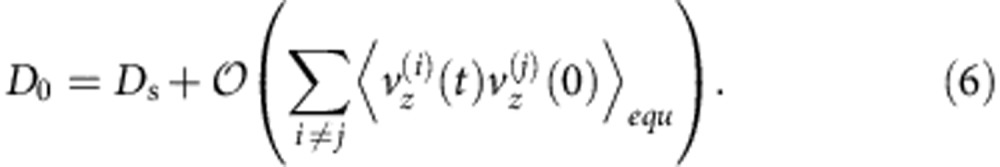


**Figure 4:**
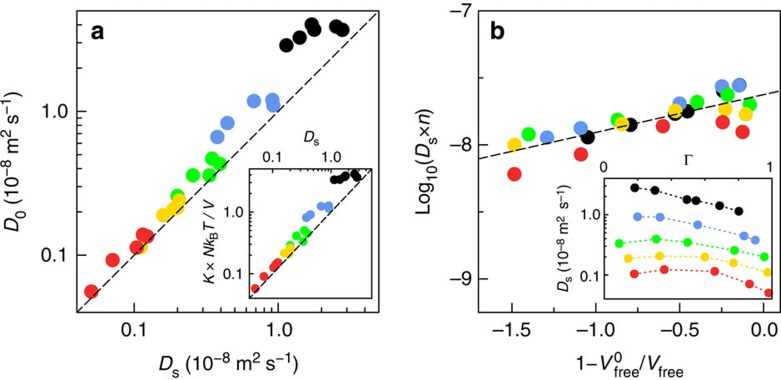
Mass transport and diffusion: towards a free volume theory. (**a**) Comparison of the self (*D*_s_) and collective (*D*_0_) diffusion. Inset: Scaling of the permeance *K* with the self-diffusion. (**b**) Rescaled diffusion coefficient *D*_s_ × *n*, with the alkane length *n*, versus the free volume fraction 
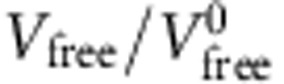
. The latter is calculated independently for a given adsorbed amount Γ, see Supplementary Information. The dashed line is the prediction of the free volume theory, 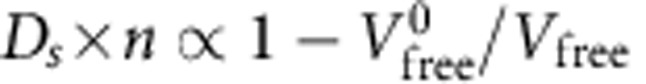, see text. Inset: bare data for the self-diffusion coefficient for the various alkanes. The colour code is the same as Fig. 1: methane (black), propane (blue), hexane (green), nonane (yellow) and dodecane (red).

In all studied systems, the difference between *D*_0_ and *D*_*s*_ was found to be small in most conditions. This is highlighted in Fig. 4a showing that *D*_0_≈*D*_*s*_, despite some differences for the shortest alkanes. Consequently, we can relate the permeance *K* to the mobility of single molecules as


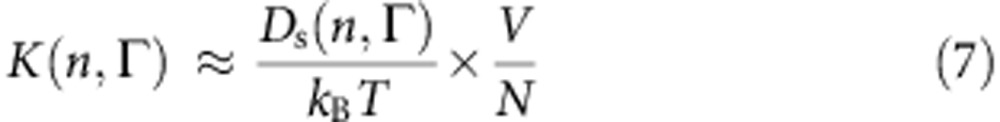


which captures the main behaviour of the permeance *K* (see the inset of Fig. 4a).

**Figure 5:**
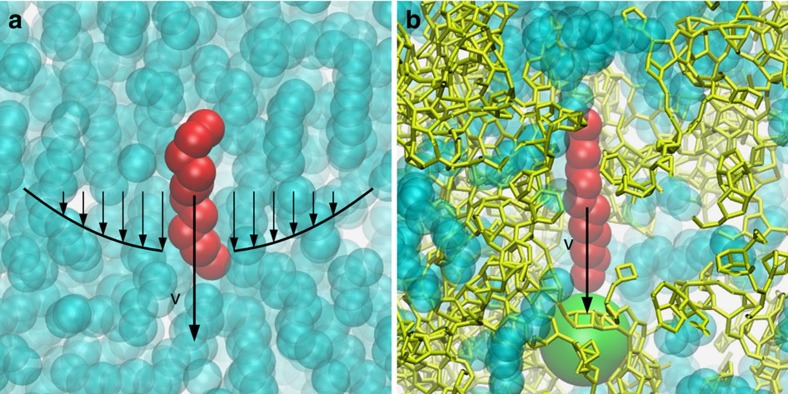
Diffusion mechanisms for bulk alkanes and alkanes confined in the porous carbon matrix. Left: In bulk, molecular diffusion is well described by the hydrodynamic Stokes–Einstein relation *D*_s_=*μk*_B_*T*=*k*_B_/(4*πηR*_0_) (with slip boundary conditions). The effective particle diameter 2*R*_0_ is consistent with 
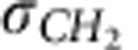
—independently of the alkane length—because the diffusive motion is mostly in longitudinal direction. Right: In contrast, in the nanopores, movement of alkane molecules is dominated by friction on the carbon matrix, corrected for the free volume accessible to the molecule (green sphere). The total friction force is a sum of the forces between the individual monomers with the pore wall—therefore scaling linearly with the alkane length. v stands for the molecule velocity.

To proceed further, one needs to provide a molecular description of the self-diffusion of the dense alkane phase in the kerogen matrix. First, we compared the scaling of the self-diffusion coefficient *D*_s_ with the chain length *n* for bulk and confined alkanes. For the bulk fluid, we find that the diffusion of a linear alkane molecule is well described by the Stokes–Einstein relation with slip boundary conditions 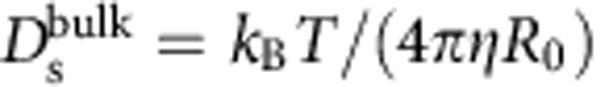 for a particle with an effective diameter close to its longitudinal cross-section 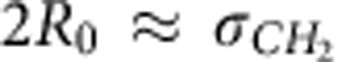; see Supplementary Fig. 6, where *R*_0_∼*D*_*s*_ × *η* is shown to be independent of the alkane density and length (under typical shale reservoir conditions *T*=423 K and *P*=25 MPa, the self-diffusivity and viscosity in the bulk liquid scale roughly as *D*_s_∼*n*^−0.7^ and *η*∼*n*^0.7^, respectively, and the *n*-dependence of the self-diffusion coefficient and the viscosity compensate each other). The fact that the hydrodynamic molecular sizes of alkanes are *independent* of their length *n* is in agreement with experimental measurements^31^. However, we emphasize that the origin of this behaviour is far from trivial. It can be actually accounted for by the slippage of the continuum alkane fluid on an individual alkane molecule along its length, as illustrated in Fig. 5a^32^. This would require further investigation of the chain dynamics using, for example, recently developed diffusion maps^33^.

Coming back to the molecular diffusion of an alkane chain in the amorphous carbon matrix, we find a very different picture, as sketched in Fig. 5b. First, as shown in Fig. 4b, we find that, like the permeance, *D*_s_ can be rescaled as the inverse of the alkane length, *D*_s_∼1/*n*. Furthermore, the rescaled diffusion *n* × *D*_s_ is found to be a generic function of the free volume accessible to the alkane molecules (Fig. 4b). Qualitatively, this scaling behaviour can be attributed to two effects. First, the strong molecular interaction of the alkanes with the carbon matrix leads to a large fluid-wall friction (in contrast to the low liquid–liquid friction in the bulk). This suggests a description in the spirit of the Rouse model for polymer diffusion^34^. Consider a single alkane molecule in the matrix. Each monomer *i* of the alkane experiences a slip-like friction force from the matrix, *f*_*v*_=−*ξ*_0_*v*_*i*_, on top of internal forces (*ξ*_0_ is the friction coefficient for a single monomer). Therefore, the total external force acting on an alkane molecule scales as *F*_*T*_=*ξ*_0_∑_*i*_*v*_*i*_=*n* × *ξ*_0_*v*, with its centre of mass velocity *v*=*n*^−1^∑_*i*_*v*_*i*_ and its mobility *μ*_*T*_=(*n* × *ξ*_0_)^−1^. Accordingly, the self-diffusion coefficient should scale as


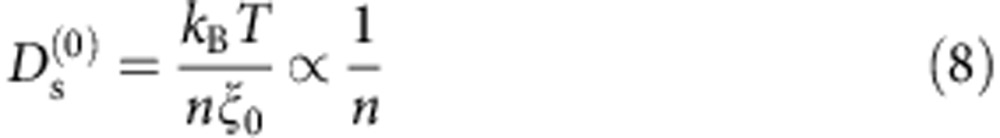


where the superscript (0) stands for a single molecule.

Now, one should take into account density effects by considering that a molecule is able to diffuse provided it finds a free cavity around it. This effect can be quantified by a free volume approach^35^,^36^, which relies on the probability to find a void space larger than a critical volume *v*_crit_ next to the diffusing molecule. One can therefore write


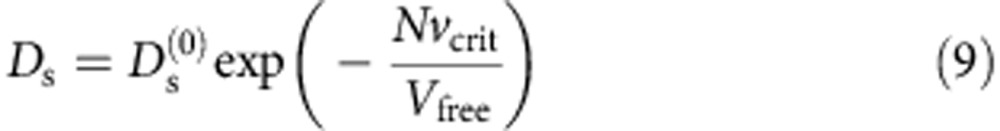


where *v*_crit_ is the minimum size of a void that allows the molecule to move into it, whereas *N* is the number of alkane molecules and *V*_free_ the accessible free volume. Estimating *v*_crit_ as the size of one alkane molecule *v*_alkane_ and using 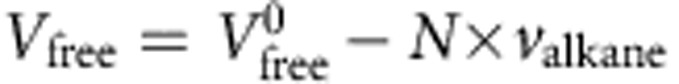 (with 
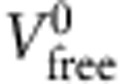
 the bare free volume in the matrix), equation (9) can be recast as:


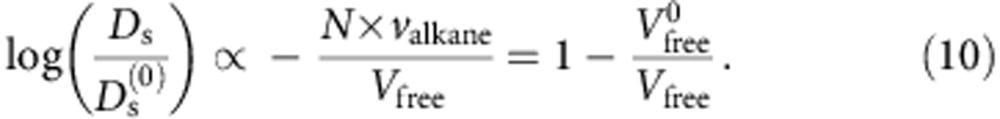


with *D*_s_^(0)^ ∝ 1/*n*. The free volume fraction 
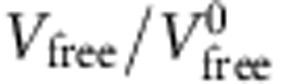
 is calculated independently for a given adsorbed amount Γ, see Supplementary Information. As shown in Fig. 4b, equation (10) describes very well the dependence of *D*_s_ on the alkane chain length and free volume, and therefore confirms the validity of our description (the calculation of the free volume in the simulations is described in the Supplementary Methods).

Altogether, these results show that the bulk and confined diffusion of alkanes follow very different mechanisms. As illustrated in Fig. 5, the molecular self-diffusion in bulk alkanes involves molecular motion that is mainly a translational movement in the longitudinal direction, subject to very little friction with the surrounding fluid molecules. In contrast, the diffusion of the confined alkanes stems merely from the friction of the molecule against the matrix, corrected for the free volume accessible to the molecule under motion.

**Figure 6:**
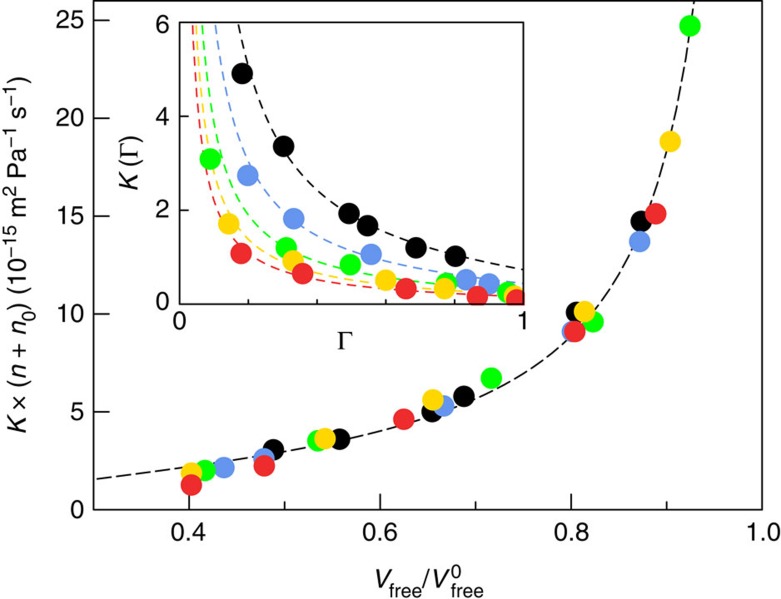
Mass transport of alkanes in nanoporous matrix. Rescaled permeance *K* × (*n*+*n*_0_) for different *n*-alkanes as a function of the free volume fraction 
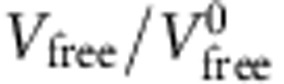
 (with *n*_0_=2). The colour code is the same as Fig. 1: methane (black), propane (blue), hexane (green), nonane (yellow) and dodecane (red). The dashed line is the prediction in equation (11), written here as 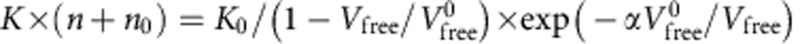, with *K*_0_=2.38 × 10^−15^ m^2^ Pa^−1^ s^−1^, *α*=0.23, obtained from a best fit. Inset: Permeance *K* versus the loading 
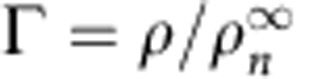
 for various alkanes. The dashed lines correspond to the previous prediction in terms of the loading, using the relationship 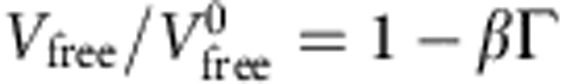, with *β*=0.6, see Supplementary Fig. 7.

Coming back to alkane transport, one therefore predicts that the permeance *K* behaves as


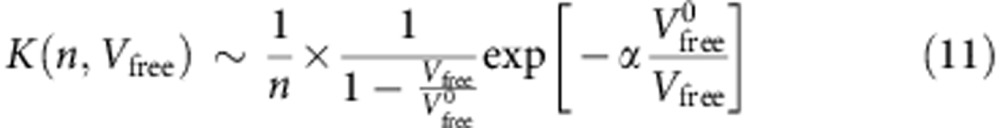


with 
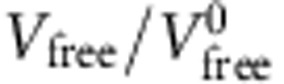
 the free volume fraction and we used the relationship 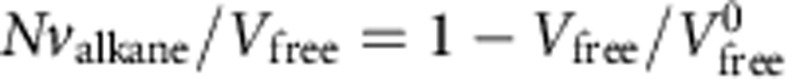; *α* is a numerical constant. Figure 6 shows that the prediction in equation (11) is in excellent agreement with the MD results for the permeance *K* for all alkanes at various densities. We allow for a shift *n*_0_ in the alkane length dependence, as our arguments provide merely the generic scaling behaviour. All the parameters needed in the derivation of equation (10) can be determined from simple experiments. 
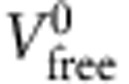
 and *V*_free_ at a given adsorbed amount *N* can be estimated from the adsorption isotherm. *α* and *D*_s_(0), which describe the dynamics of the confined alkanes, can be assessed from diffusion experiments such as Quasi-Elastic Neutron Scattering.

Finally, the permeance as described by equation (11) can be recast to describe the dependency on the loading Γ, which is an experimentally accessible quantity. One expects a linear relationship between the free volume fraction and the loading Γ, that is, 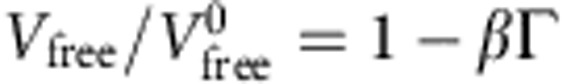. This is confirmed by our simulations, see Supplementary Fig. 7, providing the value *β*=0.60. Accordingly, one obtains the following prediction for the mass transport permeance *K* in terms of alkane length *n* and adsorbed amount Γ:


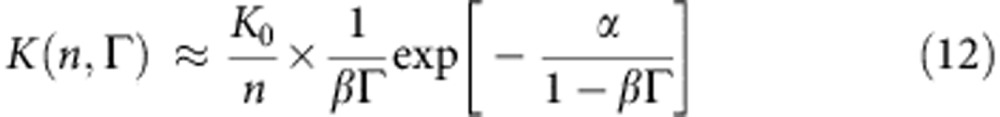


As shown in the inset of Fig. 6, this single expression provides a very good description of the permeance *K* for *all alkanes*, at *all densities* and does confirm the relevance of the underlying microscopic description. This expression takes into account both the strong adsorption of the alkane in the microporous kerogen, via the dependence on loading Γ, as well as the specific nanofluidic transport of this dense alkane phase in the disordered matrix. It provides an explicit prediction for the permeance *K*, which quantitatively captures hydrocarbon transport in the nanoporous kerogen matrix, in spite of the breakdown of Darcy's law. Furthermore, our prediction allows rationalizing the 1/*n* rescaling of the permeance, as found in Fig. 3b. Altogether our prediction, equation (12) therefore establishes a framework able to describe quantitatively hydrocarbon transport in ultra-low permeable materials.

## Discussion

We demonstrated that hydrodynamics and, hence, Darcy's law fail to describe hydrocarbon transport in nanoporous media because of strong molecular adsorption leading to non-viscous flow. As an alternative to the continuum Darcy's description, we propose a microscopic description for the permeance *K* derived from the theoretical framework of statistical mechanics, which culminates in a quantitative prediction of the permeance as a function of alkane length *n* and adsorbed amount Γ, an experimentally accessible quantity^37^. This relation offers a valuable tool for the fluid-specific prediction of hydrocarbon transport properties in ultra-low permeable media such as kerogen. Once integrated into a bottom-up model of fluid transport in multiscale porous materials (using, for example, well-established homogeneization techniques), this can be the starting point for the development of a new generation of unconventional reservoir simulators. More generally, it proposed a useful framework for mass transport of dense fluids in nanoporous materials, which is pertinent to questions relevant to catalysis, energy storage and so on. Beyond the immediate practical implications, the presented results about the exotic transport in porous materials also raises new challenging fundamental questions. In particular, the cross-over between hydrodynamic to non-hydrodynamic transport in disordered nanoporous media calls for a shift of paradigm as conventional approaches—based on percolation, porosity and tortuosity concepts—38,39 do rely on continuum descriptions. The present work offers a well-grounded molecular basis to adress these questions.

## Author contributions

L.B., B.C., R.P. and F.-J.U. designed the work. K.F. performed the simulations. K.F., L.B. and B.C. analysed the data and wrote the manuscript.

## Additional information

**How to cite this article:** Falk, K. *et al*. Subcontinuum mass transport of condensed hydrocarbons in nanoporous media. *Nat. Commun*. 6:6949 doi: 10.1038/ncomms7949 (2015).

## Supplementary Material

Supplementary Information Supplementary Figures 1-7, Supplementary Table 1, Supplementary Discussion, Supplementary Methods and Supplementary References

## References

[b1] U.S. Energy Information Administration. Annual energy outlook 2014 with projections to 2040 (U.S. Energy Information Administration, DOE/EIA-0383 (2014).

[b2] Cueto-Felgueroso L. & Juanes R. Forecasting long-term gas production from shale. Proc. Natl Acad. Sci. USA 110, 19660–19661 (2013). 24277849 10.1073/pnas.1319578110 PMC3856810

[b3] Kerr R. A. Natural gas from shale bursts onto the scene. Science 328, 1624–1626 (2010). 20576864 10.1126/science.328.5986.1624

[b4] Hughes J. D. Energy: a reality check on the shale revolution. Nature 494, 307–308 (2010). 23426309 10.1038/494307a

[b5] Tollefson J. Energy - Oil boom raises burning issues. Nature 495, 290–291 (2013). 23518538 10.1038/495290a

[b6] Vidic R. D., Brantley S. L., Vandenbossche J. M., Yoxtheimer D. & Abad J. D. Impact of shale gas development on regional water quality. Science 340, 1235009 (2013). 23687049 10.1126/science.1235009

[b7] Clarkson C. R. . Pore structure characterization of North American shale gas reservoirs using USANS/SANS, gas adsorption, and mercury intrusion. Fuel 103, 606–616 (2013).

[b8] Aguilera R. Flow Units: From Conventional to Tight-Gas to Shale-Gas to Tight-Oil to Shale-Oil Reservoirs. SPE Reservoir Evaluation and Engineering SPE-132845 SPE (2013).

[b9] Monteiro P. J. M., Rycroft C. H. & Barenblatt G. I. A mathematical model of fluid and gas flow in nanoporous media. Proc. Natl Acad. Sci. USA 109, 20309–20313 (2012). 23188803 10.1073/pnas.1219009109 PMC3528606

[b10] Patzek T. W., Male F. & Marder M. Gas production in the Barnett Shale obeys a simple scaling theory. Proc. Natl Acad. Sci. USA 110, 19731–19376 (2013). 24248376 10.1073/pnas.1313380110 PMC3856843

[b11] Bazant Z. P., Salviato M., Chau V. T., Viswanathan H. & Zubelewicz A. Why fracking works. J. Appl. Mech. 81, 101010 (2014).

[b12] Clarkson C. R., Nobakht M., Kaviani D. & Ertekin T. Production analysis of tight-gas and shale-gas reservoirs using the dynamic-slippage concept. SPE J. 17, 230–242 (2012).

[b13] Sinha S. . Steady-state permeability measurements on intact shale samples at reservoir conditions - effect of stress, temperature, pressure, and type of gas. *Society of Petroleum Engineers*. doi:10.2118/164263-MS (2013).

[b14] Ortiz-Young D., Chiu H. C., Kim S., Voitchovsky K. & Riedo E. The interplay between apparent viscosity and wettability in nanoconfined water. Nat. Commun. 4, 2482 (2013). 24052015 10.1038/ncomms3482

[b15] Coasne B., Galarneau A., Pellenq R. & Di Renzo F. Adsorption, intrusion and freezing in porous silica: the view from the nanoscale. Chem. Soc. Rev. 42, 4141–4171 (2012). 23348418 10.1039/c2cs35384a

[b16] Neimark A. V. & Vishnyakov A. Phase transitions and criticality in small systems: vapor–liquid transition in nanoscale spherical cavities. J. Phys. B 110, 9403–9412 (2006). 10.1021/jp056407d 16686483

[b17] Valiullin R. . Exploration of molecular dynamics during transient sorption of fluids in mesoporous materials. Nature 443, 965–968 (2006). 17066029 10.1038/nature05183

[b18] Levesque M., Duvail M., Pagonabarraga I., Frenkel D. & Rotenberg B. Accounting for adsorption and desorption in lattice Boltzmann simulations. Phys. Rev. E 88, 013308 (2013). 10.1103/PhysRevE.88.013308 23944584

[b19] Botan A., Vermorel R., Ulm F. & Pellenq R. J. M. Accounting for adsorption and desorption in lattice Boltzmann simulations. Langmuir 29, 9985–9990 (2013). 23886335 10.1021/la402087r

[b20] Bocquet L. & Tabeling P. Physics and technological aspects of nanofluidics. Lab. Chip. 14, 3143–3158 (2014). 25046581 10.1039/c4lc00325j

[b21] Thomas J. A. & McGaughey A. J. H. Water flow in carbon nanotubes: transition to subcontinuum transport. Phys. Rev. Lett. 102, 184502 (2009). 19518876 10.1103/PhysRevLett.102.184502

[b22] Won Y. & Aluru N. R. Water permeation through a subnanometer boron nitride nanotube chang. J. Am. Chem. Soc. 129, 2748–2749 (2007). 17305343 10.1021/ja0687318

[b23] Firouzi M., Rupp E. C., Liu C. W. & Wilcox J. Molecular simulation and experimental characterization of the nanoporous structures of coal and gas shale. Int. J. Coal Geo. 121, 123–128 (2014).

[b24] Orendt A. M. . Three-dimensional structure of the Siskin Green River Oil Shale Kerogen Model: a comparison between calculated and observed properties. Energy Fuels 27, 702–710 (2013).

[b25] Kelemen S. R. . Direct characterization of kerogen by X-ray and solid-state ^13^C nuclear magnetic resonance methods. Energy Fuels 21, 1548–1561 (2007).

[b26] Pikunik J., Llewellyin P., Pellenq R. J. M. & Gubbins K. E. Argon and nitrogen adsorption in disordered nanoporous carbons: simulation and experiment. Langmuir 21, 4431–4440 (2005). 16032857 10.1021/la047165w

[b27] Barrat J.-L. & Hansen J.-P. Basic Concepts for Simple and Complex Liquids Cambridge Univ. (2003).

[b28] Jain S. K., Pellenq R. J. M., Pikunic J. P. & Gubbins K. E. Molecular modeling of porous carbons using hybrid reverse Monte Carlo method. Langmuir 22, 9942–9948 (2006). 17106983 10.1021/la053402z

[b29] Smit B. & Maesen T. L. M. Molecular simulations of zeolites: adsorption, diffusion, and shape selectivity. Chem. Rev. 108, 4125–4184 (2006). 18817356 10.1021/cr8002642

[b30] Todd B. D. & Hansen J. S. Nonlocal viscous transport and the effect on fluid stress. Phys. Rev. E 78, 051202 (2008). 10.1103/PhysRevE.78.051202 19113118

[b31] Iwahashi M., Yamaguchi Y., Ogura Y. & Suzuki M. Dynamical structures of normal alkanes, alcohols, and fatty acids in the liquid state as determined by viscosity, self-diffusion coefficient, infrared spectra, and 13C NMR spin-lattice relaxation time measurements. Bull. Chem. Soc. Jpn 63, 2154–2158 (1990).

[b32] Han Y. . Brownian motion of an ellipsoid. Science 314, 626–630 (2006). 17068256 10.1126/science.1130146

[b33] Ferguson A. L., Panagiotopoulos A. Z., Debenedetti P. G. & Kevrekidis I. G. Systematic determination of order parameters for chain dynamics using diffusion maps. Proc. Natl Acad. Sci. USA 107, 13597–13602 (2010). 20643962 10.1073/pnas.1003293107 PMC2922286

[b34] Rouse P. E. A theory of the linear viscoelastic properties of dilute solutions of coiling polymers. J. Chem. Phys. 21, 1272–1280 (1953).

[b35] Cohen M. H. & Turnbull D. Molecular transport in liquids and glasses. J. Chem. Phys. 31, 1164–1169 (1959).

[b36] Thran A., Kroll G. & Faupel F. Correlation between fractional free volume and diffusivity of gas molecules in glassy polymers. J. Polym. Sci. B Polym. Phys 37, 3344–3358 (1999).

[b37] Kärger J. & Valiullin R. Mass transfer in mesoporous materials: the benefit of microporous diffusion measurement. Chem. Soc. Rev. 42, 4172–4197 (2013). 23377106 10.1039/c3cs35326e

[b38] Scholz C. . Permeability of porous materials determined from the Euler characteristic. Phys. Rev. Lett. 109, 264504 (2012). 23368569 10.1103/PhysRevLett.109.264504

[b39] Berkowitz B. & Ewing R. P. Percolation theory and network modeling applications in soil physics. Surveys Geophys 19, 23–72 (1998).

