# Supplementary material for: Subcontinuum mass transport of condensed hydrocarbons in nanoporous media
Source: Nat Commun. 2015 Apr 22;6:6949. doi: 10.1038/ncomms7949 (PMC4421809; doi:10.1038/ncomms7949)
Supplement: Supplementary Information — Supplementary Figures 1-7, Supplementary Table 1, Supplementary Discussion, Supplementary Methods and Supplementary References [file ncomms7949-s1.pdf]

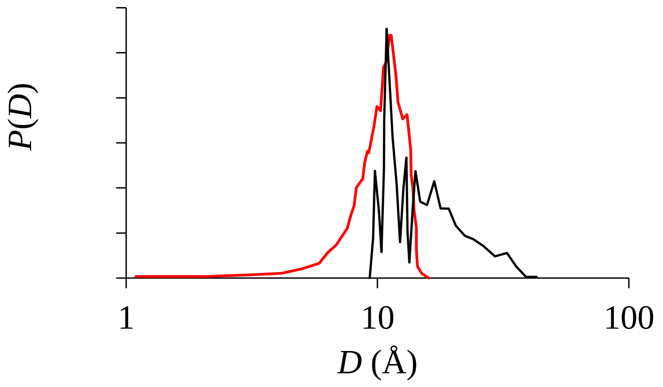


Supplementary Figure 1: Pore size distribution $P(D)$ for the numerical sample considered in this work (red line) and for an experimental kerogen sample (Marcellus kerogen, black line). The experimental data, which are taken from Clarkson et al. Fuel (2013), were obtained by means of $CO_2$ adsorption.

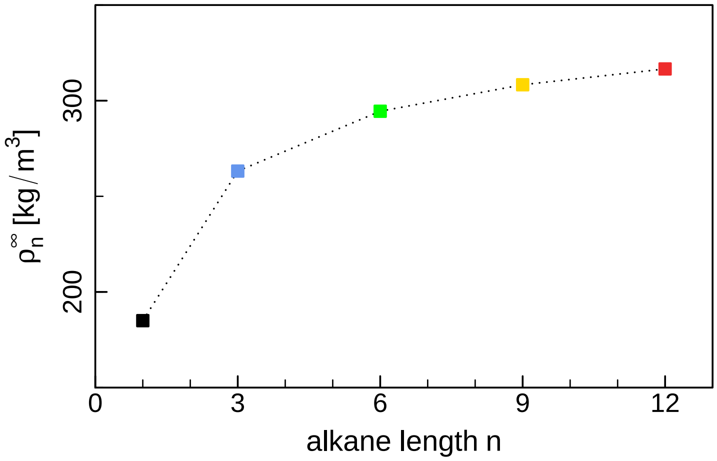


Supplementary Figure 2: Maximum density $\rho_n^\infty$ of methane (black), propane (blue), hexane (green), nonane (yellow) and dodecane (red) that can be adsorbed in CS1000a. The values for $\rho^\infty$ were obtained from a Langmuir-fit on the adsorption isotherms (see also Fig. 1c in the article).

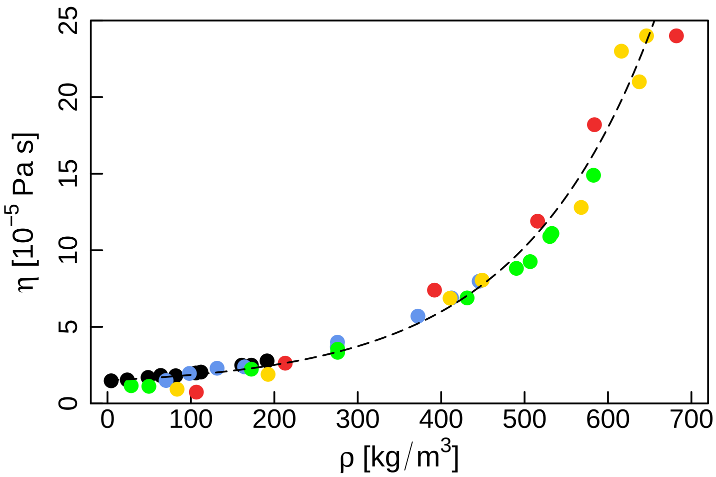


Supplementary Figure 3: Bulk viscosity $\eta$ of different *n*-alkanes (colors as in Supplementary Figure 1) as a function of the fluid mass density $\rho$. These values were obtained in equilibrium bulk MD simulations from the Green-Kubo formula for the viscosity Eq. (7). The dashed line is a fit of the form $A\exp(B\rho) + C$.

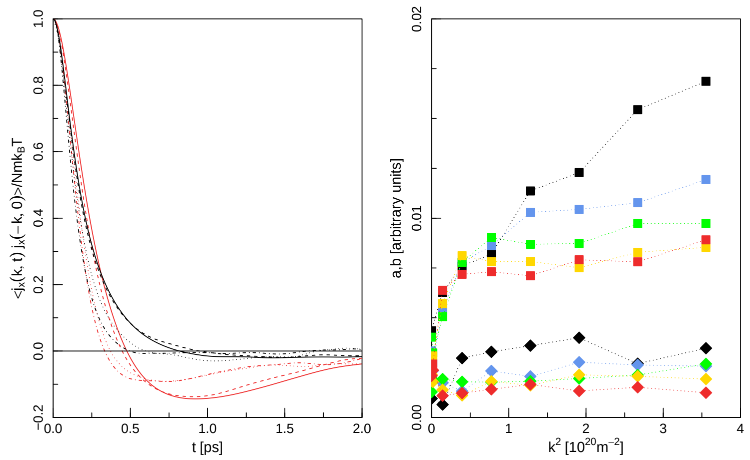


Supplementary Figure 4: Transverse current autocorrelation function for methane (black) and dodecane (red) for $4$ different $k$-values ($k = 0, 0.13, 0.38, 0.68$ Å$^{-1}$, full, dashed, dotted and dash-dot line, respectively) *(left)*. The form of the transverse current autocorrelation function is totally different from the expectation for a simple viscous fluid. The transverse current correlations do not follow a simple exponential decay, but a double exponential of the form $A\exp(-at) - B\exp(-bt)$, and the decay coefficients do not scale with $k^2$ *(right)*.

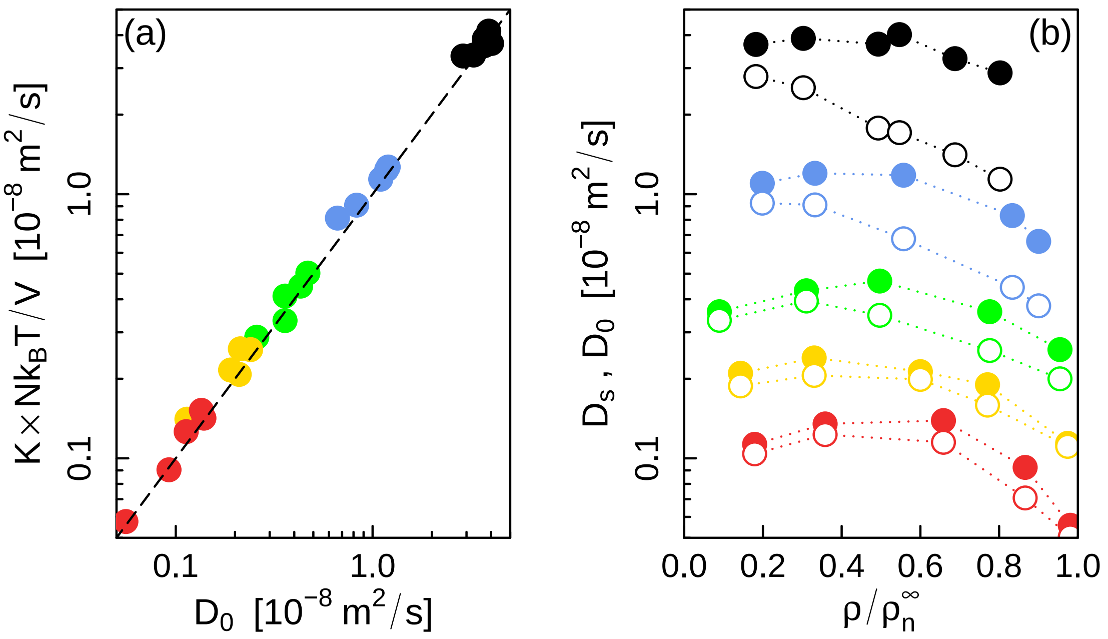


Supplementary Figure 5: *(a)* Permeance $K$ measured in steady state flow simulations *vs.* collective diffusion coefficient measured in equilibrium MD simulations, and *(b)* comparison of the collective and the self diffusion coefficients as a function of loading $\Gamma = \rho/\rho^{\infty}$. Note the logarithmic scale of the $y$-axis. Colors denote different alkane lengths (as in Supplementary Figure 1).

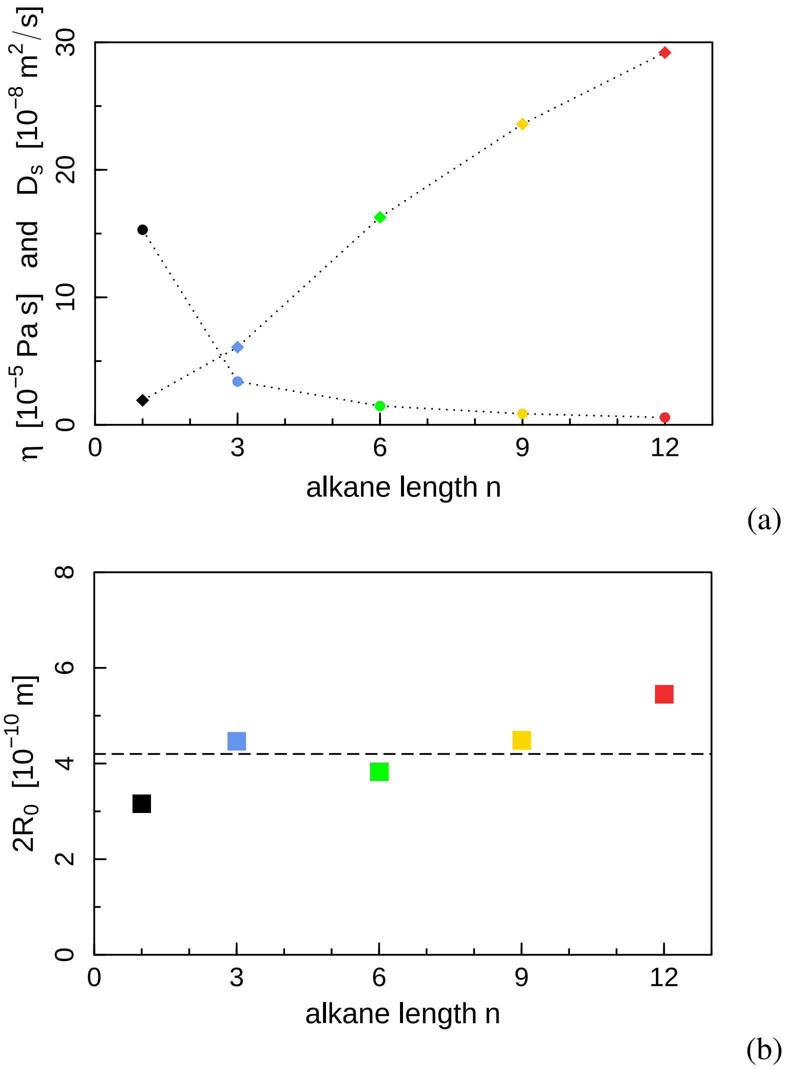


Supplementary Figure 6: Viscosity and self diffusion *(a)*, as well as effective molecular diameter $2R_0$ *(b)* of different $n$-alkane bulk fluids at $T = 423\,\mathrm{K}$ and $P = 25\,\mathrm{MPa}$. The dashed line in (b) marks the LJ size $2^{1/6}\sigma$.

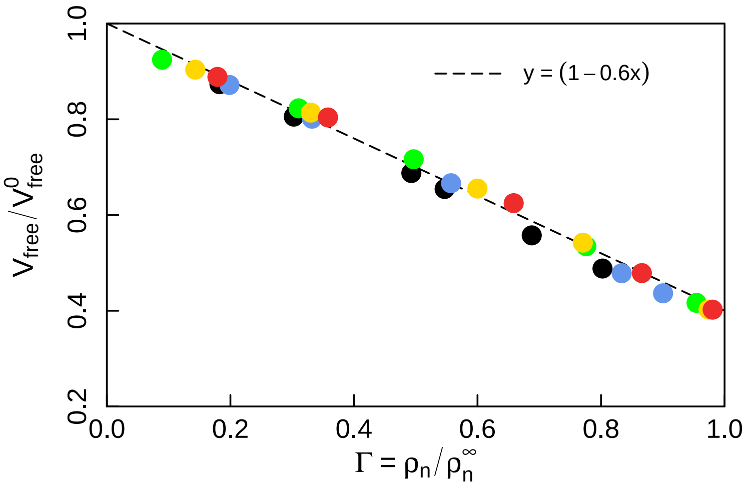


Supplementary Figure 7: Conversion between the loading $\Gamma$ and the free volume fraction $V_{free}/V$ for all considered *n*-alkanes (colors as in Supplementary Figure 1). The loading was determined in grand canonical Monte Carlo adsorption simulations (see Supplementary Methods), and the free volume was calculated numerically by simple random sampling (see Supplementary Methods).

Supplementary Table 1: Lennard-Jones parameters for carbon and the different functional groups in the alkane chains. Parameters for interactions between different types are calculated from the Lorentz-Berthelot mixing rules (see text).

| atom or group type | $\varepsilon/k_B$ (K) | $\sigma$ (Å) |
|---|---|---|
| $CH_4$ | 148 | 3.73 |
| $CH_3$ | 98 | 3.75 |
| $CH_2$ | 46 | 3.95 |
| $C_{CS1000a}$ | 28 | 3.36 |

## 1 Supplementary Discussion

### Adsorption isotherms

The adsorption isotherms in Fig. 1c of the article were obtained in CB-GCMC simulations of alkanes in the carbon matrix at different fugacities (equivalent to chemical potential $f = f_0 \exp(\mu/k_B T)$, that is $\mu VT$-ensemble). To give the adsorption isotherms in terms of pressure instead of fugacity, the fugacity-pressure relation was determined by comparing bulk simulations in the $\mu VT$ (CB-GCMC) and the $NPT$ (MD) ensemble at the same density according to the method reported in [6].

The adsorption isotherms can be described by a Langmuir form

$$\rho(f) = \rho^{\infty} \frac{\kappa f}{1 + \kappa f} \tag{1}$$

where $\rho^{\infty}$ is the maximum possible density of alkanes, which is reached in the limit $f \to \infty$ and $\kappa$ is connected to the isosteric heat of adsorption $Q_{st}$. Both $\rho^{\infty}$ and $Q_{st}$ depend systematically on the alkane length $n$:

- The isosteric heat of adsorption in the low pressures limit $Q_{st}^0$ scales linearly with the alkane length, because every monomer contributes equally.
- The maximum mass density increases slightly with the alkane length (see Supplementary Figure 2). In other words, the pore space can be filled a little denser with larger alkanes. This somewhat counter-intuitive result is due to the relatively high flexibility of the alkanes,

and to the fact that the intramolecular distance between two functional groups is smaller than for two groups of different molecules.

The continuous form of the adsorption isotherms shows that no gas-liquid transition (i.e. capillary condensation) occurs. For methane and propane this is expected, since $T = 423\,\mathrm{K}$ is above the critical temperature for both fluids. This is not the case for the longer alkanes however, where under the same thermodynamic conditions bulk fluid undergoes a phase transition. The dependence of phase behavior on confinement, i.e. in interface dominated systems, is a well-known effect.[9]

## Molecular dynamics simulations

**Linear response.** A linear relationship

$$q = -K\nabla P \tag{2}$$

was observed for all cases, that is to say for all considered $n$-alkanes (up to dodecane), pressure gradients (up to $\sim 10^{16}$ Pa/m) and flow directions. As it is typically the case in MD simulations, the pressure gradients were far larger than in realistic systems, excluding non-linear effects in a far larger range than the relevant range of pressure gradients. Concerning the flow direction, the driving force was generally applied along the $z$-direction of the carbon matrix. But for one dense system of dodecane, flow in all three dimensions was investigated and compared. No differences were observed for the transport properties. This is expected because the structural characteristics of the CS1000a model are isotropic.

**Non-Darcy behavior.** Based on Darcy's law, the permeance $K = -q/\nabla P$ is expected to behave like $k/\eta$, where $\eta$ is the fluid viscosity and $k$ a material constant of the matrix – its permeability. In other words, the product $K \times \eta$ should be constant, independently of the fluid type, thermodynamic conditions and applied pressure gradient. Fig. 2 in the article shows that this is not the case here. In order to describe the fluid flow with a Darcy-like expression we would have to assume a fluid and density dependent permeability. One reason for this is the different phase behavior of the confined fluid (see previous discussion of adsorption properties). In the traditional description, adsorption effects are not taken into account. The viscosity of the fluid inside the pores is assumed to be the same as for a bulk fluid under the same thermodynamic conditions. However, the density of fluids in nanopores can be considerably different than in bulk. Therefore, one might consider taking these adsorption effects into account by using the viscosity of the fluid phase in the pores. However, the definition of viscosity in this disordered and confining pore network is highly non-trivial,[11] which is the reason why we abstain from calculating a *confined* fluid viscosity. Instead, we make a first estimate from equilibrium simulations of bulk fluid at the same temperature and density as in the pores (NVT ensemble) using the Green-Kubo[10] relation

$$\eta = \frac{V}{k_B T} \int_0^\infty \langle P_{ij}(t) P_{ij}(0) \rangle_{equ} \, \mathrm{d}t \tag{3}$$

with $P_{ij}$ an off-diagonal element of the stress tensor. This procedure implies that the simulated bulk phase is not strictly at the same pressure as the confined fluid. Interestingly, we find that the temperature and pressure dependence of the viscosity is small compared to the influence of density, meaning that fluids at different $(T, P)$-conditions, but at the same $\rho$, will have very similar

viscosity – independently of the alkane length. As shown in Supplementary Figure 3, the viscosity can be estimated according to

$$\eta(\rho) = A\exp(B\rho) + C \tag{4}$$

with $A = 0.41$, $B = 6.2 \times 10^{-3}$ and $C = 1.1$ ($\rho$ in kg/m$^3$ and $\eta$ in $10^{-5}$ Pa s). However, in the end, this modified Darcy approach does not correctly describe the transport properties either (see inset of Fig. 3a in the article).

**Transverse current.** We consider the momentum current $\mathbf{j} = \sum_l m\mathbf{v}^{(l)}$ ($m$ is the mass of one alkane molecule, and $\mathbf{v}^{(l)}$ the velocity of molecule $l$). The transverse current time autocorrelation function (in Fourier space)

$$\langle j_z(k_x,t)j_z(-k_x,0)\rangle_{equ} \quad \text{with} \quad j_z(k_x,t) = \sum_l mv_z^{(l)}(t)\exp(-\mathrm{i}k_x x^{(l)}(t)) \tag{5}$$

offers some valuable information about the dynamic behavior of a fluid. ¿From the Navier-Stokes equation for a simple incompressible fluid

$$\rho\partial_t\mathbf{v} = -\nabla P + \eta\nabla^2\mathbf{v} \tag{6}$$

it follows analytically that the long-time behavior of the transverse current autocorrelation function is an exponential decline

$$\langle j_z(k,t)j_z(-k,0)\rangle_{equ} = Nmk_BT\exp\left(-\nu k^2 t\right) \tag{7}$$

with a decay parameter that depends quadratically on the mode $k$, and linearly on the kinematic viscosity $\nu = \eta/\rho$. Adsorbed inside a solid matrix, we would then expect a relaxation of the

form $\exp\left[-\left(\gamma_0 + k^2\nu\right)\right]$, where $\gamma_0$ stems from the friction of the liquid with the solid matrix. This hydrodynamic behavior is found for none of the studied systems. Instead of the simple exponential decay, we find a double exponential of the form

$$\langle j_z(k,t) j_z(-k,0)\rangle_{equ} = A\exp(-at) - B\exp(-bt) \tag{8}$$

and the coefficients $a$ and $b$ do not scale with $k^2$, but converge to constant values for large $k$ (see Fig. S4). This is a clear indication that the *n*-alkane dynamics cannot be described by the classical hydrodynamics equations for a simple viscous fluid. Note that the observed form of the transverse current autocorrelation function does not agree with a visco-elastic behavior either. The deviations from the expected exponential form are more pronounced with increasing alkane length, hinting at the confinement as the origin of the failure of the classical hydrodynamic theory in the studied systems.

**Self and collective diffusion.** More information about the macroscopic transport properties can be obtained from studying diffusion processes. Indeed the permeance can also be expressed in the form

$$K = \frac{D_0}{\rho_N k_B T} = \frac{V}{3k_B T}\int_0^\infty \langle q(t)\cdot q(0)\rangle_{equ}\,\mathrm{d}t \tag{9}$$

with $D_0$ a collective diffusion coefficient. The second equality is the fluctuation dissipation theorem for the collective diffusion, relating $D_0$ to the time correlation function of the velocity fluctuations of the fluid center-of-mass with respect to the (frozen) carbon matrix $q(t) = \frac{1}{N}\sum_l \mathbf{v}^{(l)}$ (with $\mathbf{v}^{(l)}$ the velocity of molecule $l$). The factor 3 stems from averaging over three coordinates, which is possible since the transport properties were found to be isotropic. The above relation offers

an independent possibility to determine the permeance in equilibrium MD simulations. Fig. S5(a) confirms that results from both methods, equilibrium fluctuations and steady-state flow, agree very well. The collective diffusivity is related to the molecular self diffusivity

$$D_s = \frac{1}{3N}\int_0^\infty \sum_l \langle \mathbf{v}^{(l)}(t)\cdot\mathbf{v}^{(l)}(0)\rangle_{equ}\,\mathrm{d}t \tag{10}$$

as follows:

$$D_0 = D_s + \frac{1}{3N}\int_0^\infty \sum_{l,j(l\neq j)} \langle \mathbf{v}^{(l)}(t)\cdot\mathbf{v}^{(j)}(0)\rangle_{equ}\,\mathrm{d}t\,. \tag{11}$$

It turns out that the remaining cross-correlation part (the integral in Eq. (15)) is often small compared to the self-correlation part $D_s$, as can be seen in Fig. S5(b) and Fig. 4(a) in the article.

The self diffusion of one alkane molecule in a bulk fluid is in agreement with the Stokes-Einstein relation with slip conditions

$$D_s^{bulk} = \frac{k_B T}{4\pi\eta R_0} \tag{12}$$

for a particle with effective diameter $2R_0$. Putting in the values for the self diffusion and the viscosity (obtained in equilibrium MD simulations, see discussion above), we find that the effective diameter for the whole alkane chain is about the size of one monomer $2R_0 \approx 2^{1/6}\sigma_{CH_2} \approx 4.2$Å (shown in Fig. S6), as is expected[12]. Hence, contrary to the confined case, the length of the alkanes does not play an explicit role for the diffusion in bulk. The origin of the $n$-dependence of the diffusion in the matrix is the interaction of the alkane chain with the matrix, which scales with the length.

### Free volume calculation

In order to estimate the free volume $V_{free}$ that appears in Fig. 4b and Eqs. (8-10) in the article, we performed a numerical calculation of the void space between hard spheres with diameter $\sigma$ (see Table 1) for representative alkane/CS1000a configurations at different loadings $\Gamma$. This calculation of the void space (=free volume) was done by randomly choosing a large number of sample points $\mathcal{N}$ (typically $\mathcal{N} = 50000$) inside the simulation box, and checking for each of them if the coordinates lie inside a sphere with $\sigma/2$-radius around any solid or fluid atom, or not. In the latter case, the point is counted as lying in the void. The free volume is estimated from the ratio of the number of points in void space to the total number of sample points: $V_{free}/V = \mathcal{N}_{void}/\mathcal{N}$.

## 2 Supplementary Methods

### Molecular Models

In order to assess the transport and adsorption properties of *n*-alkanes in amorphous nanoporous carbons, we performed molecular dynamics (MD) and Grand Canonical Monte Carlo simulations with an insertion and configuration bias (CB-GCMC), respectively. In the following, we describe the models that were used for the alkane molecules and for the carbon matrix.

**Porous carbon structure.** A realistic model of pyrolized (at $1000^\circ$ C) and activated saccharose was used for the carbon structure. The atomistic structure of this model, referred to as CS1000a, was constructed by Jain *et al.* with the Hybrid Reverse Monte Carlo method to match experimental X-ray diffraction data. It is thus representative of the real sample[1,2]. The CS1000a

model contains 4526 carbon atoms in a volume of $(5\,\mathrm{nm})^3$. The pore size distribution ranges from about $0.3$ to $1.3\,\mathrm{nm}$, and all pores are interconnected. The pore accessibility for hydrocarbons was tested in a combined approach of MD and CB-GCMC simulations: we compare the fluid density in the CS1000a matrix found in grand canonical MC simulations with the fluid density found after imbibition from an outside bulk reservoir. Supplementary Figure 1 shows that the pore size distribution for this numerical sample spans from a few Å to $\sim$ 15Å, which is consistent with the pore sizes probed by $CO_2$ adsorption in kerogen.

**Alkane model.** The *n*-alkanes were modeled with a united atom force field [3]. Bonds between two coarse grained beads and bending between two neighboring bonds are constrained by harmonic potentials $U(X) = 0.5C_X(X - X_0)^2$ ($X = b, \theta$). The equilibrium bond length and inter-bond angle are $b_0 = 1.54$Å and $\theta_0 = 114°$. Flexibility of the alkane chains is further constrained by the torsion potential

$$U_{torsion}(\phi) = C_1\,(1 + cos(\phi)) + C_2\,(1 - cos(2\phi)) + C_3\,(1 + cos(3\phi)) \tag{13}$$

with $C_1/k_B = 355.03\,K$, $C_2/k_B = -68.19\,K$ and $C_3/k_B = 791.32\,K$ ($k_B$ is the Boltzman constant). Non-bonded liquid-liquid and liquid-solid interactions are modeled with the Lennard-Jones (LJ) potential

$$U_{LJ}(r_{ij}) = 4\varepsilon_{ij}\left(\left(\frac{\sigma}{r}\right)^{12} - \left(\frac{\sigma}{r}\right)^{6}\right) \tag{14}$$

where $r_{ij} = |\mathbf{r_i} - \mathbf{r_j}|$ is the distance between the two interacting atoms. The parameters $\varepsilon_{ij}$ and $\sigma_{ij}$ are calculated with the Lorentz-Berthelot mixing rules $\varepsilon_{ij} = \sqrt{\varepsilon_i \varepsilon_j}$ and $\sigma_{ij} = \frac{1}{2}(\sigma_i + \sigma_j)$,[4] and the LJ parameters $\varepsilon_i$ and $\sigma_i$ are given in Supplementary Table 1. The positions of the carbon atoms

in the CS1000a structure are frozen. Consequently, the results for the alkane flow rates presented in this study are lower limits, because the alkane molecules might deform a flexible structure and thereby increase their mobility. We assume that the effect of flexibility is a systematic one, and that it is small compared to the influence of the alkane size.

### Configurational biased Grand Canonical Monte Carlo simulations

Adsorption simulations were performed with the Grand Canonical Monte Carlo technique ($\mu VT$ ensemble). Monte Carlo steps consisted of translation, rotation, partial regrowth, insertion and deletion of alkane molecules. Due to the relatively large size of the molecules (up to dodecane) and the high density of the system (pore diameters of the order of the molecular size), a biased procedure was used for the insertion and for the regrowth of molecules in order to enhance the probability to find an energetically favorable configuration. Otherwise, the acceptance rate would be impractically low. The applied bias procedure follows the scheme introduced by Smit and can be found in [5].

### Molecular Dynamics

The MD simulations were performed with the LAMMPS package[7]. Integration of the equations of motion was performed with a 1 fs time step. Simulations were performed in the NVT ensemble. Initial configurations with different alkane densities were taken from the CB-GCMC adsorption simulations. The pressures corresponding to these densities were obtained from a combination of GCMC and MD simulations of bulk fluids. A Nosé-Hoover thermostat with a relaxation time

of $0.1\,\mathrm{ps}$ was applied to keep the temperature of the fluid at $423\,\mathrm{K}$, a typical value for unconventional reservoirs (From a practical point of view a high temperature has the advantage to accelerate the particle dynamics and thus to equilibrate the system faster). For the non-equilibrium simulations the center of mass velocity was subtracted from the atom velocities before the rescaling. In some simulations with low fluid density the thermostat was applied to a relatively small number of molecules (around 30), which might result in a spurious effect of the thermostat on the flow rate. We checked that this is not the case by replicating the dodecane system with the lowest density in every dimension (eight times larger volume) and repeating measurements. No influence of the system size on the fluid transport properties was observed.

Flow rates were measured for different driving forces by applying an external gravitational field g of different magnitude. Applying a constant acceleration g is representative for different setups where flow is induced by an external driving force: In particular, it is equivalent to applying a pressure gradient

$$\nabla P = -\rho \mathbf{g} \tag{15}$$

where $\rho$ is the fluid density. Or, instead of a pressure gradient, the driving force could also be a chemical potential gradient – both are related by the Gibbs-Duhem equation $\rho_N \mathrm{d}\mu = \mathrm{d}P$ at constant temperature, as was also verified in simulations by Arya et al. [8] In the following, results are presented in terms of pressure gradients, in order to make a direct connection to the extraction of hydrocarbon fluids from an underground reservoir, and to Darcy's law. ¿From the steady-state

flow simulations, we obtain a value for the mean fluid flow velocity

$$q = \frac{1}{N} \sum_l v_z^{(l)} \tag{16}$$

in the carbon matrix. We studied this mean flow velocity as a function of the driving force (i.e. pressure gradient), the alkane length, and the fluid density. The temperature was kept constant at $423\,\mathrm{K}$.

## Supplementary References


1. S. K. Jain, Pellenq, R. J. M., Pikunic, J. P. & Gubbins, K. E. Molecular modeling of porous carbons using hybrid reverse Monte Carlo method, *Langmuir* **22**, 9942-9948 (2006).

2. Pikunik, J., Llewellyin, P., Pellenq, R. J. M. & Gubbins, K. E. Argon and nitrogen adsorption in disordered nanoporous carbons: simulation and experiment, *Langmuir* **21**, 4431-4440 (2005).

3. Martin, M. G. & Siepmann, J. I. Transferable potentials for phase equilibria. 1.United-atom description of n-alkanes, *J. Phys. Chem. B* **102**, 2569-2577 (1998).

4. Allen, M. P. & Tildesley D. J., *Computer simulations of liquids* (Clarendon Press, Oxford, 1989).

5. Smit, B. Grand-canonical Monte Carlo simulations of chain molecules: Adsorption isotherms of alkanes in zeolites, *Mol. Phys.* **85**, 153-172 (1995).

6. Desbiens N., Boutin A. & Demachy, I. Water condensation in hydrophobic Silicalite-1 Zeolite: A molecular simulation study, *J. Phys. Chem. B* **109**, 24071-24076 (2005).

7. Plimpton, S. Fast parallel Algorithms for short-range molecular dynamics. *J. Comp. Phys.* **117**, 1-19 (1995); http://lammps.sandia.gov.

8. Arya G., Chang H. -C. & Maginn E. J., A critical comparison of equilibrium, non-equilibrium and boundary-driven molecular dynamics techniques for studying transport in microporous materials, *J. Chem. Phys.* **115**, 8112-8124 (2001).

9. Coasne, B., Galarneau, A., Pellenq, R. & Di Renzo, F. Adsorption, intrusion and freezing in porous silica: the view from the nanoscale, *Chem. Soc. Rev.* **42**, 4141-4171 (2012).

10. Kubo, R. The fluctuation-dissipation theorem, *Rep. Prog. Phys.* **29**, 255-285 (1966).

11. Todd B.D. & Hansen J.S., Nonlocal viscous transport and the effect on fluid stress, *Phys. Rev. E* **78**, 051202 (2008).

12. Iwahashi M., Yamaguchi Y., Ogura Y. & Suzuki M., Dynamical structures of normal alkanes, alcohols, and fatty acids in the liquid state as determined by viscosity, self-diffusion coefficient, infrared spectra, and 13C NMR spin-lattice relaxation time measurements, *Bull. Chem. Soc. Japan* **63**, 2154-2158 (1990).
